# Carrier immobilization and auto-exposition favoring reuse of silyletherase SilE-R from *Brassica* sp. with high activity and enantiospecificity

**DOI:** 10.1007/s10529-025-03600-9

**Published:** 2025-05-29

**Authors:** Lisa Pick, Anna L. Schumacher, Elif Öztel, Thorsten Mascher, Marion B. Ansorge-Schumacher

**Affiliations:** 1 Chair of Molecular Biotechnology, Dresden University of Technology, Dresden, Germany; 2 Chair of General Microbiology, Dresden University of Technology, Dresden, Germany

**Keywords:** Biocatalysis, Carrier immobilization, Repetitive process, Silylether hydrolysis, SporoBead

## Abstract

**Objectives:**

Investigation of immobilization methods promoting the use of silyletherases from *Brassica* sp. for the efficient and enantiospecific hydrolysis of silyl-protected hydroxyl functions.

**Results:**

Different supports for adsorptive and covalent binding of the silyletherase SilE-R as well as exposure of the enzyme on the surface of *Bacillus subtilis* endospores, so-called SporoBeads, were evaluated. While the highest protein loading of 26 mg enzyme per gram was obtained by adsorptive binding, the best combination of specific activity and enantiospecificity was obtained when SilE-R was exposed on SporoBeads. Protein loading was estimated at 2.6 mg per gram of spore, which was in the same range as after covalent binding to a carrier. In six repeated reaction cycles, SporoBeads exposing SilE-R lost less than 10% of their catalytic activity. The enantiomeric excess could not be increased even with short reaction times, but remained constant over all repeated cycles.

**Conclusion:**

The exposure of silyletherases on SporoBeads has been identified as a promising approach for the synthetic application of this novel type of enzyme, although some properties relevant for catalytic applications need to be further improved.

**Supplementary Information:**

The online version contains supplementary material available at 10.1007/s10529-025-03600-9.

## Introduction

Silylethers represent a class of compounds that occupy an important position in the field of organic chemistry, particularly in the context of multi-step synthesis of complex molecules. This is due to their ability to effectively protect hydroxyl functions from undesired reactions (Kocienski 2005). Recently, two enzymes were identified for the first time that are capable of specifically and enantioselectively hydrolyzing silylether bonds (Pick et al. 2024). Thus, they provide the fundamental possibility of combining protection and kinetic resolution of racemic alcohols in a biocatalyzed reaction, thereby intensifying the synthesis process.

The two known silyletherases, SilE-R and SilE-S, are highly homologous molecules with opposing enantiopreferences. The respective preference can be influenced by molecular manipulation, but the complete enantiospecificity that would be optimal has not yet been achieved. The maximum enantiomeric excess of the product is currently 70%, with a decrease observed with increasing concentration of the non-preferred substrate enantiomer. Therefore, the biotechnological use of the enzymes for kinetic racemic resolution is favored by a high concentration of the racemic substrate, necessitating continuous or repeated reaction approaches. The feasibility of such approaches is contingent upon the ability to retain the enzymes within the reactor or to recover them subsequent to the reaction. The optimal method for achieving this is through immobilization.

Immobilization captures enzymes in a specific phase, on or in a carrier material so that they can be easily separated from the product (Ansorge-Schumacher 2024). This greatly facilitates the practical handling and reuse of biocatalysts. In view of the high proportion of enzyme costs in biocatalytic processes, this also increases the economic efficiency of the processes. The identification of suitable immobilization methods is therefore an important aspect in the development of enzyme-catalyzed processes, and numerous methodological approaches have been described, including adsorption, covalent binding, embedding and encapsulation, as well as combined processes. Each of these approaches has advantages and disadvantages; catalytic activity is usually reduced, while stability can be significantly increased. It is rarely possible to predict which immobilization approach will give the best results for a particular catalyst and reaction type.

By far the most widely used immobilization approach for enzymes is adsorptive or covalent binding to the surface of solid supports. Various materials are commercially available for this purpose. With these materials, immobilization is relatively easy, the materials have good mechanical robustness, and restrictions on mass transfer are comparably low. The latter favors high catalytic activity, especially in the conversion of large substrates and compounds with low water solubility and therefore low concentration. Both features are highly relevant in protection chemistry with silylethers. The covalent binding is characterized by high strength, while the mobility of the enzymes and thus their catalytic activity can be significantly limited. In contrast, the catalytic activity of adsorbed enzymes is often higher, while larger amounts of enzymes are lost over time due to desorption.

An interesting alternative to the well-established covalent binding of enzymes to solid supports is autoimmobilization by coupling enzymes to proteins exposed on the surface of biological systems (Lozancic et al. 2019). This involves coupling the enzyme to a natural linker molecule during production in the host organism and then directing this construct to the surface where it is permanently located. This combines active production and immobilization, eliminates the need for enzyme purification, avoids potential negative effects of chemically linking enzymes to a carrier, and, as an added benefit, eliminates the cost and waste of an external carrier. A well-known system using this concept is phage display, in which the gene encoding the target enzyme is cloned into the genome of a filamentous phage (Soumillion et al. 1994). However, this method only allows the display of relatively small proteins. Display on the surface of microbes such as *Escherichia coli*, *Bacillus* spec., *Staphylococcus* spec. and *Saccharomyces cerevisiae* via fusion with an anchoring motif (Lee et al. 2003) as well as on endospores of *Bacillus subtilis* (Wang et al. 2017) has also been described. A major challenge in the use of vegetative cells is the requirement that target proteins must (at least partially) cross the cell membrane for display. This requires post-translational processing, and steric hindrance, incomplete exposure, or misfolded/unfolded structures can severely compromise catalytic activity (Vaart et al. 1997). *Bacillus* endospores do not cause this problem because the assembly of the spore takes place inside the cells. The spores also combine good expression capacity with low pathogenicity, high thermal and storage stability, and short fermentation times (Wang et al. 2017). Thus, they display promising capacity for biocatalysis. Bartels et al. (2018) demonstrated good biocatalytic activity of two laccases on *Bacillus* endospores after fusion with the crust proteins CotY and CotZ. For the active spores they established the term “Sporobeads”.

**Scheme 1:**
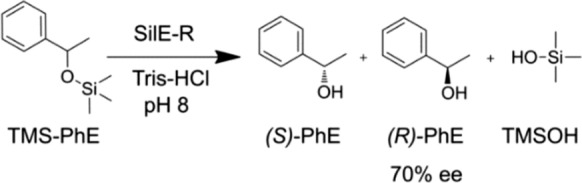
Hydrolysis of trimethylsilanol-protected phenylethanol (TMS-PhE) to phenylethanol (PhE) and trimethylsilanol (TMSOH). *Ee* Enantiomeric excess of *(R)*-PhE over *(S)*-PhE catalyzed by free SilE-R (Pick et al. 2024)

The objective of this study was to identify an immobilization method for silyletherases that ensures high apparent specific activity with maximum enantiospecificity and allows for repeated synthetic applications. A range of commercially available supports with varying material properties were chosen for non-covalent and covalent binding. For practical and economic reasons, display on SporoBeads was also evaluated using CotY as a fusion protein. Given its superior accessibility, SilE-R was selected as the model enzyme and the hydrolysis of trimethylsilanol-protected phenylethanol (TMS-PhE) was investigated as the standard reaction (Scheme 1).

## Materials and methods

**Fig. 1:**
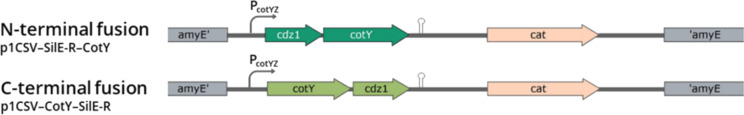
Map of integrative vector segment with N- or C-terminally fused *cdz1* coding for SilE-R. The segment shown is the part of the sporovector that integrates into the amyE locus of the *B. subtilis* BKE 3013 genome. It consists of the strongest crust operon promoter PcotYZ, *cdz1* fused to *cotY*, a transcriptional terminator and the *cat* gene for chloramphenicol resistance

Carriers IB-S60P, IB-A369, Cov-5, Cov-7 and Cov-9 were obtained from ChiralVision (The Netherlands), Lewatit VP OC 1600 from Lanxess (Germany), Celite R632 from Imerys Filtration Minerals (France) and Accurel MP1001 from Membrana GmbH (Germany). Chemicals for substrate synthesis, buffers and media were purchased from Sigma Aldrich (Germany), except TMSCl, which was purchased from Thermo Fisher Scientific (Germany). Solvents were purchased from Roth (Germany). Soluble SilE-R (Uniprot accession: M4CDZ1) was produced and purified as described previously (Pick et al. 2024); for trimethylsilanol-protected phenylethanol (TMS-PhE) hydrolysis, the preparation had a specific activity of 298 U g_prot_^−1^. SporoBeads were derived from three different strains of *B. subtilis* BKE 3013, which contains an *mls* cassette encoding resistance to erythromycin and lincomycin in place of the native *lipC* gene. Two of the strains contained *cdz1* encoding SilE-R fused to cotY (Uniprot accession: Q08311) and a *cat* gene encoding chloramphenicol resistance integrated through *amyE* (Fig. 1). In one of these strains SilE-R was fused to the N-terminus of CotY and in the other strain it was fused to the C-terminus. The third strain was a control without the *cdz* gene.

### Binding to solid supports

For non-covalent immobilization, all selected carrier materials were incubated in a micro test tube with pure ethanol for 30 min at 40 rpm on a horizontal rotator/roller mixer RM5.6 (M. Zipperer GmbH, Germany) and at room temperature. The ethanol was then removed with a pipette; the adsorptive binding supports were washed twice with Milli-Q water and the ionic support was washed twice with Tris/HCl buffer (pH 8). The carriers were then incubated for 4 h with buffered (100 mM Tris/HCl pH 7.5, 150 mM NaCl) enzyme solution containing 5 mg SilE-R per 100 mg carrier at 40 rpm on the horizontal rotator RM5.6. For covalent immobilization, SilE-R was incubated with the selected supports as described above, except that the supports were not pretreated with ethanol. After incubation, the enzyme solutions were removed with a pipette and the protein concentration was determined using Nanodrop ND1000 (ε1% 9.1, λ = 280 nm; Thermo Fisher Scientific Inc., USA). All carriers were washed once with Milli-Q water. Subsequently, the carriers were dried in a desiccator over silica gel orange (Roth, Germany) for two to three days.

### Production of SporoBeads

Precultures of the CotY-SilE-R fusion protein strains were grown on LB medium with chloramphenicol (5 μg mL^−1^), lincomycin (25 μg mL^−1^), and erythromycin (5 μg mL^−1^). The medium of the control strain was LB with only lincomycin (25 μg mL^−1^) and erythromycin (5 μg mL^−1^). Precultures were incubated overnight at 37 °C and 180 rpm. 1 L of Difco Sporulation Medium was inoculated with 2 mL of the overnight culture and grown for 24 h at 37 °C and 180 rpm. Cell harvest was performed by centrifugation at 10,000 g, 10 min and room temperature. All subsequent centrifugation steps were performed consistently. For cell lysis, each pellet was treated with 75 μg lysozyme mL^−1^ in 10 mL deionized H_2_O at 37 °C for 1 h. After centrifugation, the pellet was washed with 10 mL deionized H_2_O by resuspending, centrifuging, and discarding the supernatant. The pellet was then washed with 10 mL of 0.05% sodium dodecyl sulfate, followed by three additional washes with 10 mL of deionized H_2_O. To obtain a measurable dry weight, all spores were lyophilized. They were frozen at − 80 °C and then lyophilized at − 62 °C and 1 mbar for 2 days. The dried spores were stored at 4 °C.

### Determination of enzyme loading

The average quantity of enzyme loaded on solid supports E_carr_ was calculated from the difference in protein concentration in the enzyme solution before and after immobilization according to Eq. 1, where m_e_: total amount of immobilized enzyme [g_e_]; m_carr_: total amount of carrier used for immobilization [g_carr_]; c_0_: protein concentration before incubation with carrier [g L^−1^]; c_1_: protein concentration after incubation with carrier [g L^−1^]; V: volume of enzyme solution used for immobilization [L]. Given that a purified enzyme was employed, the enzyme concentration was deemed to be equivalent to the protein concentration. The protein concentration was determined using Nanodrop ND1000 (ε1% 9.1, λ = 280 nm; Thermo Fisher Scientific Inc., USA).

1 $${E}_{carr}= \text{ } {m}_{e}/{m}_{carr}\text{=}\left({c}_{0}-{c}_{1}\right)\cdot V/{m}_{carr}$$

The average quantity of enzyme exposed on SporoBeads E_sp_ was calculated from the catalytic activity of the spores and the free enzyme according to Eq. 2, where m_e_: total amount of immobilized enzyme [g_e_]; m_sp_: total amount of spores [g_sp_]; A_sp_: apparent specific catalytic activity of spores [U g_sp_^−1^]; A_e_: specific catalytic activity of free enzyme per mass of protein [U g_prot_^−1^]. Given that a purified enzyme was employed for the determination of A_e_, the enzyme concentration was deemed to be equivalent to the protein concentration. The protein concentration was determined using Nanodrop ND1000 (ε1% 9.1, λ = 280 nm; Thermo Fisher Scientific Inc., USA).

2 $${E}_{sp}= \text{ } {m}_{e}/{m}_{sp}\text{=}{A}_{sp}/{A}_{e}$$

The average number of enzyme molecules exposed on one spore Ne_sp_ was calculated from the spore loading E_sp_ and the spore weight according to Eq. 3, where N_e_: number of exposed enzyme molecules; N_sp_: number of spores; W_sp_: Average weight of a single spore [g_sp_]; N_A_: Avogadro number (6.022⋅10^23^ mol^−1^); MW_e_: molecular weight of one enzyme subunit, i.e. 12,321 g mol^−1^ for SilE-R (Pick et al. 2024). The calculation was performed with a single subunit of the enzyme, as each CotY-anchor molecule was fused with only one subunit.

3 $${Ne}_{sp}\text{=}{N}_{e}/{N}_{sp}\text{=}{E}_{sp}\cdot {W}_{sp}\cdot {N}_{A}/{MW}_{e}$$

The average weight of a single spore W_sp_ was determined from the spore concentration and number in a suspension according to Eq. 4, where N_sp_: total number of spores in a sample; c_sp_: spore concentration in the suspension [g_sp_ L^−1^]; V: sample volume [L]; N_count_: number of spores counted in the sample; df: dilution factor. A spore suspension with a concentration of 10 g L^−1^ (c_sp_) was diluted 1:20 with water and a sample was transferred into a Thoma cell counting chamber. A photograph was taken (Supplementary information, Fig S1), and the spores in the 16 small squares comprising the central square were counted (198 spores). The standardized volume of the 16 squares (V) is 4⋅10^–10^ L.

4 $${W}_{sp}\text{=}{m}_{sp}/{N}_{sp}\text{=}{c}_{sp}\cdot V/\left({N}_{count}\cdot df\right)$$

### Activity assays

Enzyme activity assays were performed using racemic trimethylsilyl protected 1-phenylethanol (TMS-PhE). Substrate synthesis and enzyme assays were performed as previously described (Pick et al. 2024). Briefly, all enzyme assays were performed using 50 mmol Tris/HCl L^−1^ (pH 8), substrate and other additives were added as indicated, and the reaction mixture was incubated at 400 rpm (Thermomixer compact, Eppendorf SE, Germany) at room temperature. For analysis, the entire reaction mixture was extracted with iso-hexane. Analysis of substrate and product concentrations was performed by gas chromatography on a Shimadzu GC-2010 Pro equipped with an AOC autosampler, FID, and a chiral HYDRODEX GAMMA-DiMOM column (Macherey–Nagel, Germany). Nitrogen was used as the carrier gas with a pre-column pressure of 100 kPa. 2 µL of sample were injected with a 1:30 split. The temperature program was set at 110 °C (11 min). Retention times were: TMS-PhE (4.5 min), *(S)*-1-phenylethanol: 9.2 min; *(R)*-1-phenylethanol: 10 min. For determination of specific activity, product formation was measured at three different time points at minimum using separate samples.

### Determination of enantiomeric excess

The enantiomeric excess (ee) of *(R)*-PhE over *(S)*-PhE was calculated from the concentrations of both products according to Eq. 5, where c_R_: concentration of *(R)*-PhE; c_S_: concentration of *(S)*-PhE.

5 $$ee\text{=}{c}_{R}-{c}_{S}/\left({c}_{R}\text{+}{c}_{S}\right)\cdot 100\text{\%}$$

### Determination of enzyme leaching

Leaching was estimated by incubating an enzyme-loaded support in buffer (50 mmol Tris/HCl L^−1^, pH 8) for 48 h and measuring the protein concentration in the supernatant using Nanodrop (ε1% 9.1, λ = 280 nm; Thermo Fisher Scientific Inc., USA). Catalytic activity in the supernatant was also determined.

### Determination of catalyst stability

Catalyst stability was determined as the percentage residual activity (A_res_) after incubation under defined conditions and after a defined time according to Eq. 6, where A_t_: hydrolytic activity after incubation; A_0_: hydrolytic activity before incubation or in the control.

6 $${A}_{res}\text{=}{A}_{t}/{A}_{0}\cdot 100\text{\%}$$

Storage stability of dry catalyst, catalyst in water and catalyst in buffer (50 mmol Tris/HCl L^−1^, pH 8) was determined at − 20 °C, 4 °C and 20 °C after 1 d, 45 d and 90 d incubation. Solvent stability was determined after 4 h incubation in buffer containing 10% (v/v) of dimethylsulfoxide (DMSO), dimethyl- formamide (DMF), acetonitrile (MeCN), 2- propanol (i-PrOH), 2-methyltetrahydrofuran (2-MTHF), methanol (MeOH), ethanol (EtOH), and ethyl acetate (EtOAc), respectively. Water was utilized as control. Thermal stability was determined after incubation on a thermomixer (Thermo Fisher Scientific Inc., USA) over several hours at room temperature, 30 °C, 40 °C, 50 °C, 60 °C, 70 °C, and 80 °C. Samples were taken at different time points. All measurements were performed in triplicates.

## Results and discussion

### Carrier binding

SilE-R was successfully attached to five commercial carriers designed for non-covalent binding and to three carriers designed for covalent binding (Table 1). All carriers were particulate, but differed in chemical composition, hydrophobicity, and size. One support, IB-A396, had anionic functions. In the case of covalent binding, only epoxide functions were employed as the reactive residues, as they do not necessitate a separate activation step prior to enzyme coupling (Ansorge-Schumacher 2024).

**Table 1:** Characteristic features of and SilE-R loading on commercial carriers. The material properties were obtained from the manufacturers’ instruction leaflets

	Carriers for non-covalent binding					Carriers for covalent binding		
Commercial name	IB-S60P	Lewatit VP OC 1600	IB-A396	Accurel MP1001	Celite R632	Cov-5	Cov-7	Cov-9
Basic polymer	silica	DVB^a^-crosslinked (poly) meth-acrylate	poly (styrene)	poly (propy-lene)	silica	(poly) acrylate	(poly) acrylate	silica
Hydrophobicity	hydrophilic	hydrophobic	hydrophobic	hydrophobic	hydrophilic	hydrophobic	hydrophilic	strongly hydrophilic
Functional group	–	–	NR_4_^+ b^	–	–	epoxy, hexa-decyl-/octadecyl	epoxy	propyl epoxide
Particle size [µm]	60–200	320–450	350–700	400–1000	595–1410	200–710	300–600	250–400
Loading [mg_e_ g_carr_^−1^]	25.9 ± 0.4^c^	25.7 ± 0.2^c^	25.6 ± 1.5^c^	8^d^	7.5 ± 1.3^e^	3 ± 0.2^e^	2.1 ± 0.2^e^	2 ± 0.1^e^

^a^divinylbenzene; ^b^quarternary ammonium functions; ^c^from duplicate measurements; ^d^from single measurements; ^e^from triplicate measurements

The highest protein loading was observed for the non-covalent binding of SilE-R to IB-S60P, Lewatit VP OC 1600 and IB-A396. The results were nearly identical for all three materials and approximately three fold higher than for adsorptive binding to Accurel MP1001 and Celite R632. They were almost one order of magnitude higher than for covalent binding to Cov-5, Cov-7 and Cov-9.

In the context of non-covalent binding, the base material and the hydrophobicity of the carrier material do not appear to exert a significant influence. This is demonstrated by the discrepancy in loading between Celite and IB-S60P, both of which are unmodified silica particles. In contrast, the size of the supports and thus the available surface area could exert an influence, as IB-S60P, Lewatit VP OC 1600, and IB-A396 are, on average, smaller than Accurel MP1001 and Celite R632. It is important to note, however, that Lewatit VP OC 1600 particles exhibit a high degree of dispersity. Some sources suggest that the average size is comparable to that of Accurel (Tecelao et al. 2012), which would relativize the influence of particle size. Additionally, the lowest protein loadings were observed in the covalent binding, despite the average particle size being similar to that of IB-S60P, Lewatit VP OC 1600, and IB-A396. Obviously, the density of functional groups on the material surface is the most important factor in protein loading for this type of immobilization. However, the density of the functional groups was not accessible.

### Exposure on SporoBeads

The amount of SilE-R exposed on the surface of the SporoBeads could not be directly determined. A loading of at least 2.6 mg_e_ g_sp_^−1^ was inferred from the apparent specific hydrolytic activity of the spores of 775 mU g_sp_^−1^. This loading is within the same range as that observed after covalent binding of SilE-R to the commercial carriers Cov-5, Cov-7, and Cov-9.

The calculated enzyme loading is based on the assumption that the specific activity of SilE-R bound to the spore surface is equal to the activity of the free enzyme. To the best of our knowledge, there is currently no available information regarding the effect of the crust protein CotY on the specific catalytic activity of an enzyme that has been fused with it. Furthermore, the information is not readily accessible due to the tendency of CotY to aggregate, which prevents the investigation of individual fusion proteins. However, it is established in the literature that the catalytic function of enzymes is typically altered by fusion with non-catalytically active, hydrophobic protein units (Krause et al. 2024). In the majority of cases, a reduction in activity is observed. Should the specific activity of SilE-R on SporoBeads be lower than that of the free enzyme the actual loading of the enzyme would exceed 2.6 mg_e_ g_sp_^−1^.

A reduction in the specific catalytic activity of SilE-R on the SporoBeads in comparison to its activity in a free state could also arise from the situation that the enzyme in the fusion with CotY is present in a monomeric state, whereas the free enzyme exists in a dimeric state (Pick et al. 2024). The activity of SilE-R in the monomeric state could differ from the activity in the dimeric state since the two potentially active centres per molecule might interact (Pick et al. 2024). The monomeric state of SilE-R on the crust surface is likely, since only one copy of the enzyme was fused per CotY, while it can be assumed that the spontaneous dimerization of SilE-R is rather unlikely in the assembly process of the spore crust. The assembly process is complex and relies on a network of proteins. At their core, endospores contain packed DNA that is protected by the cortex and four protein layers called the basal layer, inner coat, outer coat, and crust (Driks and Eichenberger 2016). CotY is a morphogenetic protein that forms the core structure of the outermost crust layer and was successfully used in earlier studies as an anchoring motif (Bartels et al. 2018; Shuster et al. 2019).

With a diameter of 0.8–1.2 µm (Ricca and Cutting 2003), the spores are at least 200 times smaller than the commercial carriers, resulting in a markedly larger surface area. Consequently, at the same loading, it is probable that the enzymes are present in a lower density on the spore surface than on the commercial carriers. The mean number of SilE-R molecules on the surface of a SporoBead at a minimum loading of 2.6 mg_e_ g_sp_^−1^ was determined to be 127,077, corresponding to an enzyme density on the spore surface of 10,112 µm^−2^. For the calculation, an average spore weight of 1 pg was considered, which was derived from the observed relation between spore mass and the number of spores in a given sample (Eq. 4; Supplementary Figure S1). The value is in good agreement with the weight that can be theoretically estimated from the average spore size (Ricca and Cutting 2003) and density (1.3 g cm^−3^; Dean and Douthit 1974) documented in the literature (Supplementary information, Equation S1).

Surprisingly, the exposure of SilE-R on the Sporobeads was markedly superior to that observed for laccase (BpuL) in a previous study, which reported 6000 molecules per spore following fusion with CotY (Bartels et al. 2018). This might indicate that the laccase activity was more affected than the activity of SilE-R, but could also result from a larger number of CotY molecules expressed and incorporated into the spore crust in our system. In any case, given that high display efficiency on autoimmobilization platforms has been a challenge to date (Lozancic et al. 2019; Wang et al. 2017), the number of SilE-R on the spore surface observed in this study is a highly encouraging result.

### Apparent catalytic activity and enantiospecificity of immobilizates

**Fig. 2:**
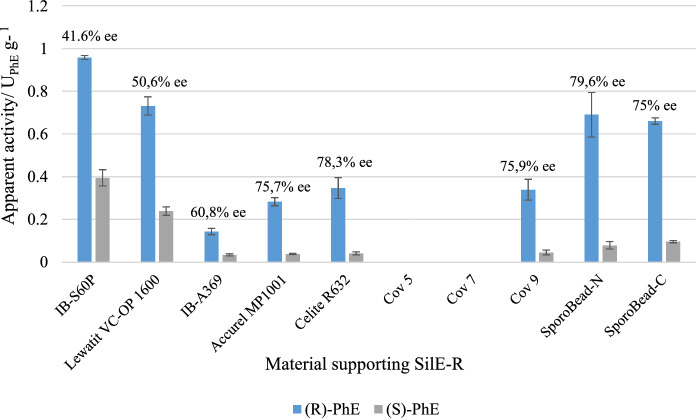
Apparent specific activity of SilE-R after adsorption to IB-S60P, Lewatit VC-OP 1600, IB-A-369, Accurel MP1001 and Celite R632, covalent binding to Cov-5, Cov-7 and Cov-9, and display on SporoBeads in N-terminal (SporoBead-N) or C-terminal (SporoBead-C) fusion with CotY. Activity refers to the amount of (*R*)-PhE and (*S*)-PhE produced by 2 mg enzyme on commercial carriers or by 0,8 mg SporoBeads in 250 µL Tris/HCl buffer (pH 8) from 5 mmol racemic TMS-PhE L^−1^ within 4 h (adsorbed enzyme and SporoBeads) or 24 h (covalently bound enzyme on commercial supports) at room temperature. Error bars indicate the standard deviation from three independent experiments. E-values were calculated based on Equation S2 and are denoted above the bars

In non-covalent binding, SilE-R demonstrated catalytic activity over the whole range of the tested carrier materials (Fig. 2). In covalent binding, the enzyme exhibited detectable activity solely in the presence of Cov-9 and on SporoBeads.

Following the initial time frame and reaction conditions, the highest apparent specific activity was observed following adsorption to IB-S60P. However, SilE-R also demonstrated the lowest enantiospecificity on this carrier: The enantiomeric excess of *(R)*-PhE over *(S)*-PhE was only 41.6%, which is markedly below the 70% reported for the free enzyme. Conversely, SilE-R showed enantiospecificity in the same range as the free enzyme when adsorbed on Accurel MP1001 and Celite R632, while only low activity was observed on these supports. The low activity might be due to the lower loading. However, despite significantly lower enzyme loading after covalent binding to Cov-9 and on SporoBeads compared to that on adsorptive carriers, the catalytic activity of SilE-R was comparable or even enhanced.

The best combination of apparent activity and enantiospecificity of SilE-R was found after display on Sporobeads. Notably, no significant difference was found between the performance of SilE-R after N- or C-terminal fusion to CotY. E-values were around 9.8 and 7.7, respectively. It is likely that this is due to the fact that SilE-R is a relatively small enzyme with its N- and C-termini in close proximity (Pick et al. 2024). Therefore, it can be reasonably assumed that fusion at either end does not lead to a significant difference in the enzyme’s activity or in the orientation on the spore surface.

**Fig. 3:**
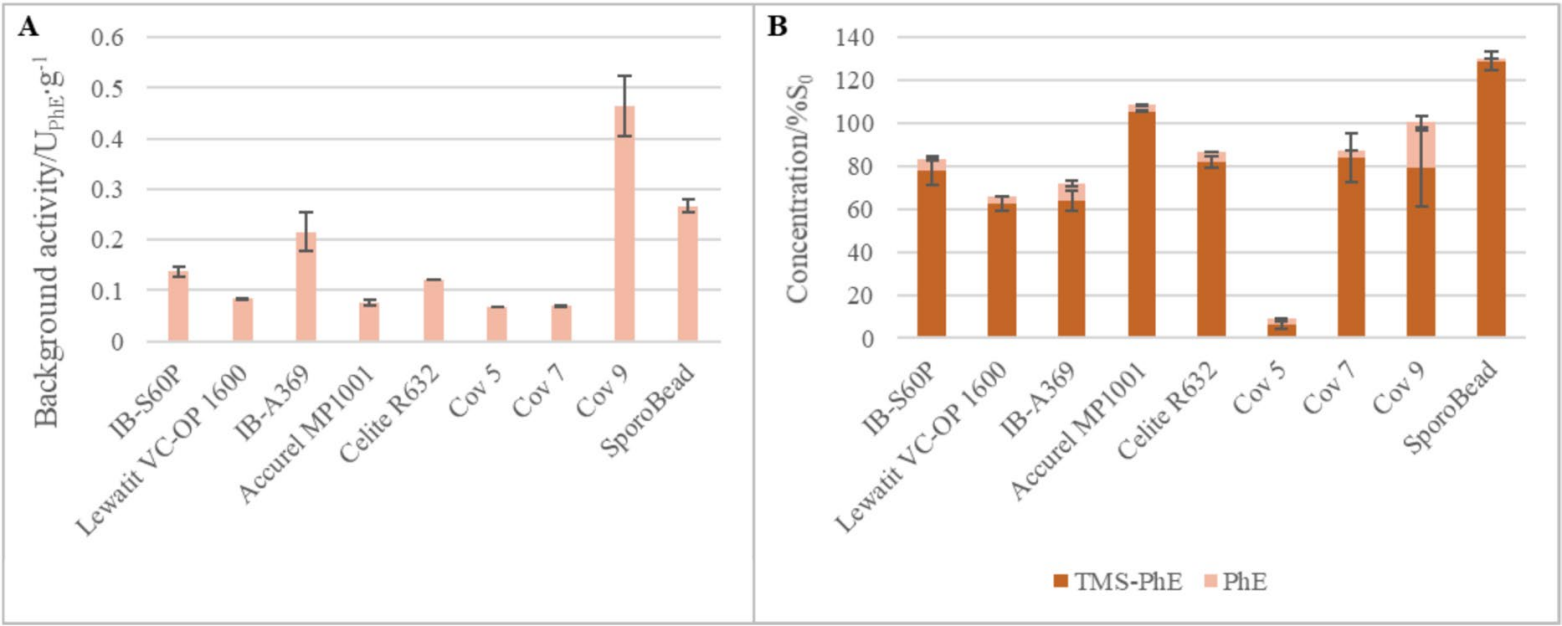
Impact of carrier material on the formation of PhE from TMS-PhE. **A** Background activity of commercial carriers and endospores from *B. subtilis*. **B** Percentual concentrations of PhE and TMS-PhE after incubation with the carrier materials. Activity refers to the total amount of PhE produced and concentration refers to the initial TMS-PhE concentration. 2 mg carrier or 0.8 mg Sporobeads were incubated in 250 µL Tris/HCl buffer (pH 8) with 5 mmol racemic TMS-PhE L^−1^ (IB-S60P, Lewatit VC-OP 1600, IB-A369, Accurel MP 1001, Celite R632), 18.5 mmol racemic TMS-PhE L^−1^ (SporoBeads) or 50 mmol racemic TMS-PhE L^−1^ (Cov-5, Cov-7, Cov-9) for 4 h (adsorption carriers and SporoBeads) or 24 h (carriers for covalent binding) at room temperature. Error bars indicate the standard deviation from three independent experiments

All experiments were conducted with consideration of the potential spontaneous hydrolysis of TMS-PhE in aqueous solution, which can also be unspecifically enhanced by non-enzymatically active material (Pick et al. 2023). Controls utilizing carrier materials or SporoBeads devoid of SilE-R exhibited background activities at a reasonable level (Fig. 3A). Spontaneous hydrolysis was notably enhanced by the hydrophilic carriers IB-S60P and Celite R632, but mostly with the charged carrier IB-A369, the carrier for covalent binding Cov-9 and the SporoBeads. The latter finding corroborates with the observation that a majority of proteins appear to facilitate TMS-PhE hydrolysis in an unspecific manner (Pick et al. 2023), and a broad array of proteins forms the surface of *Bacillus* endospores (Driks and Eichenberger 2016).

Interestingly, the background hydrolysis of TMS-PhE, which invariably leads to the formation of racemic PhE (data not shown), did not affect the enantiospecificity of SilE-R on the SporoBeads or Cov-9. Furthermore, it did not correlate with the particularly low enantiospecificity observed for SilE-R on IB-S60P and Lewatit VC-OP 1600 (Fig. 2). However, when the amount of substrate used for the experiments was compared with the sum of the spontaneously formed product and the remaining substrate after incubation, a significant mass loss was observed with IB-S60P, Lewatit VC-OP 1600 and most of the other supports (Fig. 3B). Only with Accurel MP1001, Cov-9 and SporoBeads all the substrate was retraced in the reaction medium. Except for Celite R632, the adsorption of substrate roughly correlated with the impact of the carrier on the enantiospecificity of immobilized SilE-R. Overall, the observations indicate that adsorption reduces the catalytic effect of SilE-R.

### Activity and enantiospecificity in repetitive use

**Fig. 4:**
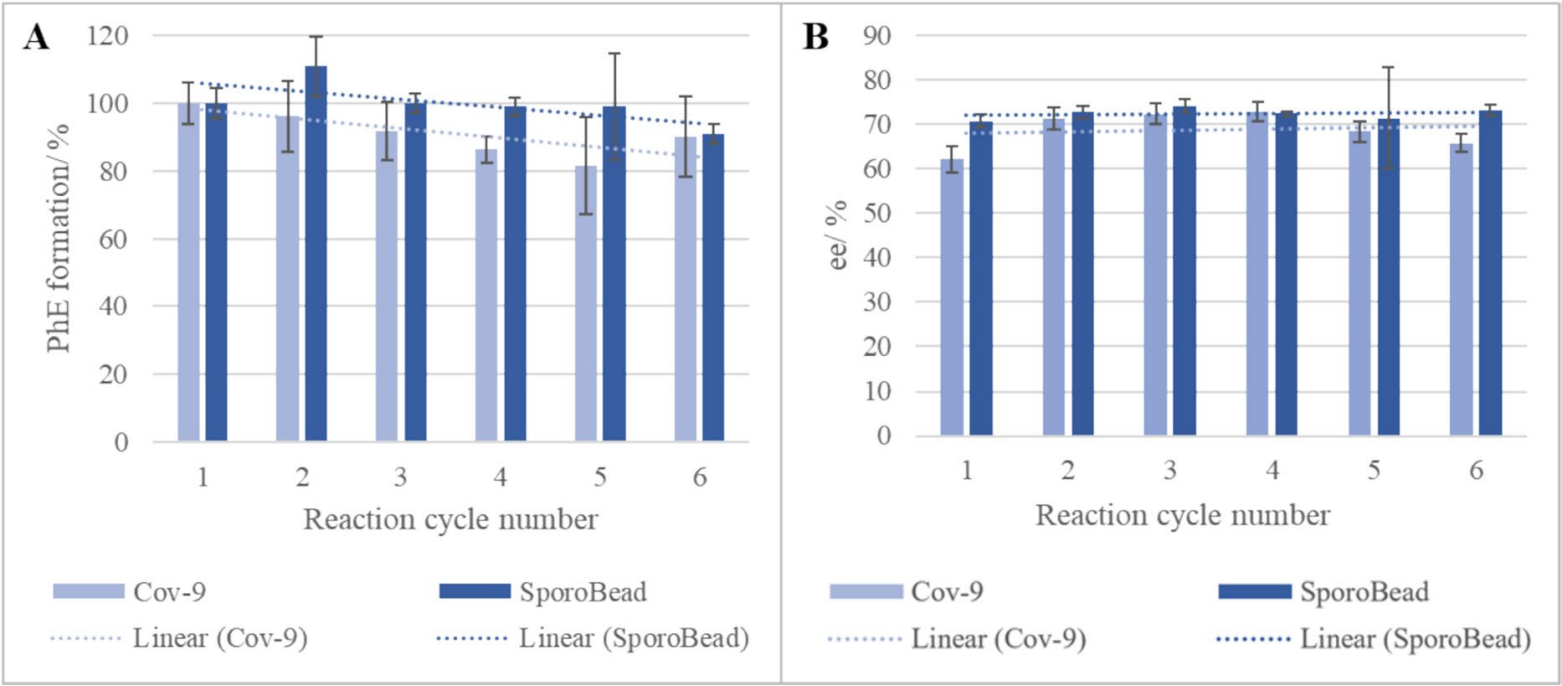
Hydrolytic activity and enantiospecificity of SilE-R immobilized on Cov-9 or exposed on SporoBeads (C-terminal fusion to CotY) upon repetitive use. Reactions were conducted in 250 µL Tris/HCl buffer (pH 8) for 30 min at room temperature using 4 mg Cov-9 with 5 mmol TMS-PhE L^−1^ (pre-dissolved in DMSO) or 2 mg SporoBeads with 18.5 mmol TMS-PhE L^−1^. After each cycle, immobilizates were removed and washed twice with buffter. **A** Percentual formation of PhE related to the product formation in the first cycle. **B** Ee of (*R*)-PhE over (*S*)-PhE. Error bars indicate the standard deviation from three independent experiments

In light of the observed catalytic activity and enantiospecificity in relation to enzyme loading, an investigation was conducted into the repeated use of SilE-R on Cov-9 and SporoBeads (Fig. 4). After six repetitive reaction cycles, a slight decrease in catalytic activity was observed, with a similar trend evident in both immobilized samples (Fig. 4A). The observed loss of activity was below 10% for SporoBeads and below 20% for Cov-9 at all times. Enzyme leaching from Cov-9 or SporoBeads was not detected (data not shown). Furthermore, the enantiomeric excess of *(R)*-PhE over *(S)*-PhE remained constant over all cycles for both immobilizates (Fig. 4B), with the average value being slightly higher when SporoBeads were used. However, an improvement in ee compared to the initial screening of the immobilizates was not observed despite the markedly shorter reaction time.

### Stability and storage of SilE-R-SporoBeads

Although the overall performance of the SilE-R immobilizates derived from Cov-9 and SporoBeads was comparable, the use of SporoBeads was superior in this study, as the same catalytic activity was achieved with half the amount of material. Consequently, additional properties of these constructs pertinent to catalytic applications were elucidated, including the impact of solvents, temperature stability, and potential storage conditions.

**Fig. 5:**
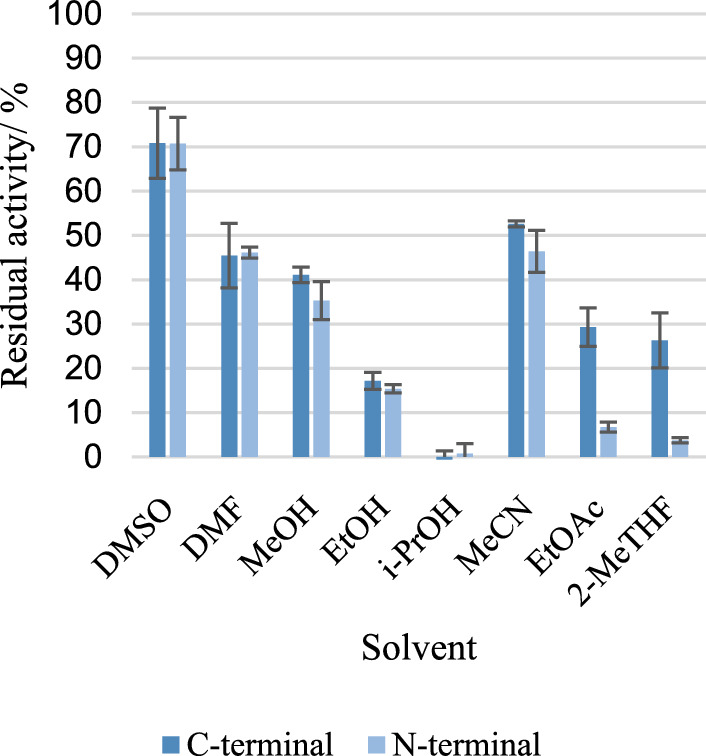
Residual hydrolytic activity of SilE-R exposed on SporoBeads (*C*- or *N*-terminal fusion to CotY) after incubation in buffer with 10% (v/v) organic solvent for 4 h. *DMSO* dimethyl sulfoxide, *DMF* dimethylfluoride, *MeOH* methanol, *EtOH* ethanol, *iPrOH*: isopropanol, *MeCN* acetonitrile, *EtOAc* ethyl acetate, *2-MeTHF* 2-methyl-tetrahydrofuran. Activity assays were conducted in 250 µL Tris/HCl buffer (pH 8) using 0.5 mg SporoBeads with 18.5 mmol TMS-PhE L^−1^. Error bars indicate the standard deviation from three independent experiments

After four hours of incubation in aqueous solutions to which a maximum of 10% (v/v) organic solvent had been added, SilE-R-SporoBeads showed a clear loss of activity (Fig. 5). The highest residual activity of about 70% of the initial activity was observed in the presence of dimethyl sulfoxide (DMSO); no residual activity was observed in the presence of isopropanol during the period under consideration. This is a significant deterioration compared to the free enzyme, which under comparable experimental conditions showed a residual activity of about 90% in the presence of DMSO and still about 25% in the presence of isopropanol (Pick et al. 2024). Interestingly, SilE-R SporoBeads generated by *C*- or *N*-terminal fusion of the enzyme with CotY behaved differently in the presence of some solvents. The *C*-terminal fusion was the more stable variant in all the cases. Very clear differences occurred with ethyl acetate (EtOAc) and 2-methyl-tetrahydrofuran (2-MeTHF), two solvents with comparatively low water solubility. The observation of these differences indicates molecular causes for the higher deactivation of the enzyme exposed on the spore surface by organic solvents compared to the free enzyme. It is possible that there is a connection with the monomeric coupling of SilE-R and CotY, which, as previously mentioned, should complicate the dimerization of SilE-R significantly. It is known that dimerization can increase the stability of enzymes (e.g. Jakoblinnert et al. 2013).

**Fig. 6:**
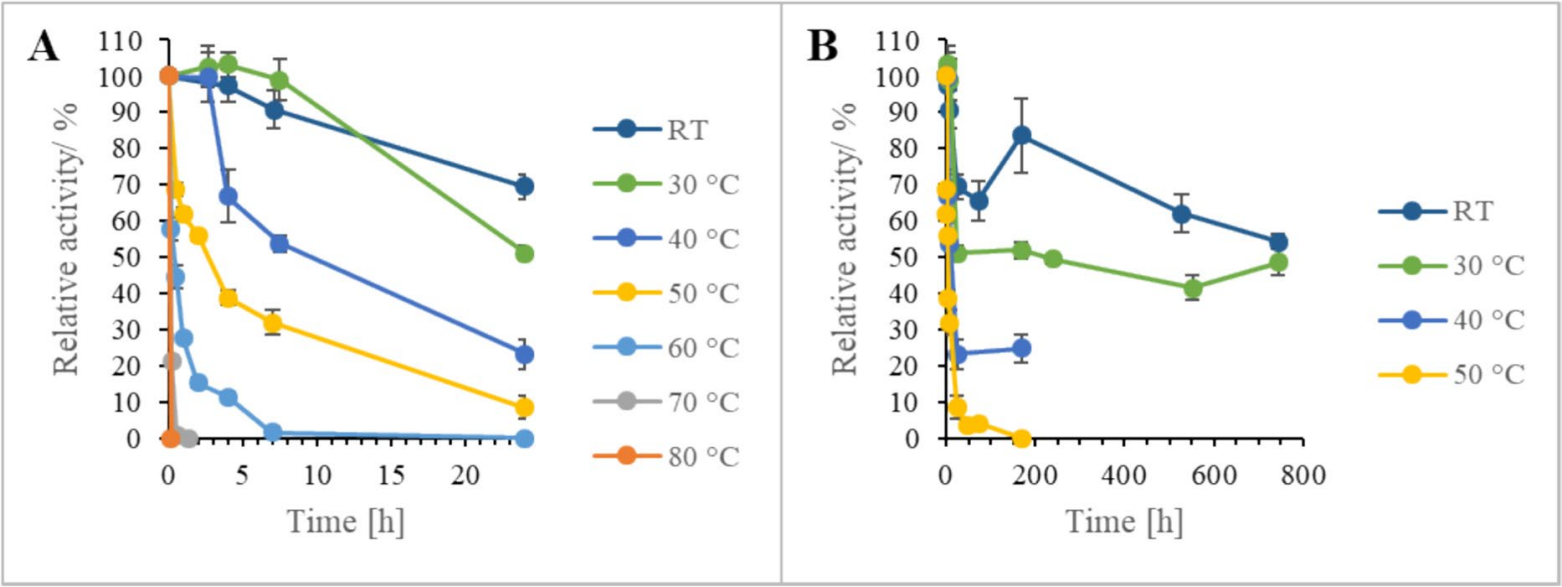
Residual hydrolytic activity of SilE-R exposed on SporoBeads (*C*-terminal fusion to CotY) after incubation in water at different temperatures. Activity assays were conducted in 250 µL Tris/HCl buffer (pH 8) using 1 mg SporoBeads with 18.5 mmol TMS-PhE L^−1^. **A** Incubation for 24 h. **B** Incubation for 30 d

SilE-R-SporoBeads showed an accelerated time-dependent loss of activity with increasing temperature (Fig. 6). Accordingly, the best stability was observed at room temperature and the worst stability at 80 °C in the experiment. This corresponds to the behavior of the free enzyme (Pick et al. 2024). Unlike the free enzyme, however, SilE-R SporoBeads exhibited a sharp drop in activity within the first 24 h (Fig. 6A), after which the loss of activity was much more moderate (Fig. 6B). At room temperature, the initial loss of activity was about 30%. Consequently, after seven days of incubation at room temperature, the SporoBeads only showed a residual activity of around 60%, while a residual activity of 90% is described for the free enzyme under comparable conditions.

For now, we can only speculate about the cause of this temperature effect on SilE-R-SporoBeads. Again, there might be a connection with the monomeric coupling of SilE-R and CotY. Should dimerization occur between fusion proteins anchored in the crust and those not bound, the initial loss of catalytic activity could indicate loose binding of the dimers and their slower or faster dissociation at different temperatures. However, a connection of the activity loss with the germination capacity of the spores would also be conceivable, since germination into a vegetative cell is accompanied by the degradation of the spore crust. Usually, germination of endospores requires the availability of nutrients (Paidhungat and Setlow 2001), which are not present in the catalytic approach. However, spontaneous germination of spore preparations in the absence of nutrients has also been reported (Paidhungat and Setlow 2000; Sturm & Dworkin 2015), so germination cannot be completely ruled out. However, it would then have to be concluded that only some of the spores reach sporulation and the remaining spores do not tend to germinate even over a longer period of time.

Storage of SilE-R-SporoBeads with a high retention of catalytic activity over a time period of 90 days was easily possible at 4 °C when the spores were dissolved in pure water, and at − 20 °C when dissolved in pure water or buffered solution (50 mmol Tris/HCl L^−1^, pH 8) or after lyophilisation (Supplementary information, Fig S2). Slight differences in storage stability were observed between SilE-R fused *C*- or *N*-terminally to CotY.

## Conclusions

The study demonstrates that SilE-R can be actively immobilized on carrier materials both adsorptively and in covalent binding. Application in repeated batch or continuous processes is therefore possible in principle. Due to the high structural similarity, it is to be expected that this can be transferred to the related silyletherase SilE-S (Pick et al. 2024).

The best results in terms of loading, catalytic activity and enantiospecificity in the hydrolysis of TMS-PhE were observed when the enzyme was covalently bound to solid supports or to the surface of SporoBeads. However, a complete loss of activity was also observed with some carriers after covalent binding, despite identical binding modalities.

From an economic and ecological point of view, the exposure of silyletherases on SporoBeads in particular offers a promising approach for biotechnological use. However, it is desirable to improve the catalytic stability of the constructs at elevated temperatures and in the presence of organic solvents, which play an important role as solubility mediators in connection with silyl ethers in protecting group chemistry (Kocienski 2005). It could possibly be achieved by generating constructs that promote the dimerization of the enzymes. However, appropriate cloning or exposure strategies have not yet been described. The use of a *B. subtilis* strain (FB85/JDB1914), which lacks the nutrient germination receptor and in which increased germination rates of the spores are therefore excluded, could also have advantages (Paidhungat & Setlow 2000; Sturm & Dworkin 2015). Due to the short reaction times, the hydrolytic application of the spores in repeated batch is already possible even without improvements. However, the hoped-for increase in enantiomeric excesses through this process approach has not yet been achieved. Further experiments must show whether this is possible by optimizing the reaction conditions.

## Supplementary Information

Below is the link to the electronic supplementary material.

Supplementary file1 (PDF 182 KB)

## Data Availability

The data that support the findings of this study will be available from the corresponding author upon reasonable request.

## Declarations

### Supporting information

**Fig. S1** Photograph of a Thoma cell counting chamber with endospore solution.

**Equation S1** Theoretical estimation of spore weight.

**Equation S2** Determination of the Enantiomeric Ratio (E-Value) for the product.

**Fig S2** Hydrolytic activity of C- and N-terminally fused SilE-R on *S*porobeads with respect to TMS-PhE hydrolysis after storage at different temperatures either dry, in water or in buffer.
