# Supplementary material for: Carrier immobilization and auto-exposition favoring reuse of silyletherase SilE-R from Brassica sp. with high activity and enantiospecificity
Source: Biotechnol Lett. 2025 May 29;47(3):59. doi: 10.1007/s10529-025-03600-9 (PMC12122602; doi:10.1007/s10529-025-03600-9)
Supplement: Supplementary file 1 — Supplementary file1 (PDF 182 KB) [file 10529_2025_3600_MOESM1_ESM.pdf]

Supplementary information

Carrier immobilization and auto-exposition favoring reuse of silyletherase SilE-R from *Brassica* sp. with high activity and enantiospecificity

Lisa Pick[1], Anna L. Schumacher[2], Elif Öztel[2], Thorsten Mascher[2], Marion B. Ansorge-Schumacher[1]*

[1]Dresden University of Technology, Chair of Molecular Biotechnology, Dresden, Germany

[2]Dresden University of Technology, Chair of General Microbiology, Dresden, Germany

*Corresponding author: marion.ansorge@tu-dresden.de; ORCID: 0000-0002-2912-546X

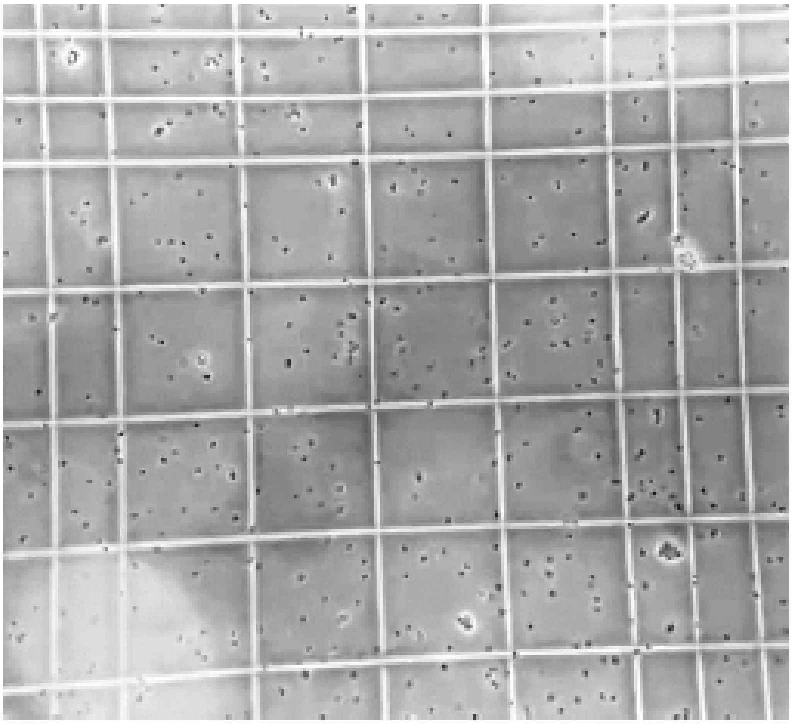

Fig S1 Photograph of a Thoma cell counting chamber with endospore solution (10 g L^{-1}, diluted 1:20).

Theoretical estimation of spore weight

The average weight of a single spore W_{sp} was estimated from literature data according to equation S1, where ρ_{sp}: average spore density [g cm^{-3}]; V_{sp}: average spore volume [cm^{-3}]; d_{sp}: average spore diameter [µm].

$$\text{(S1) } W_{sp} = \rho_{sp} \cdot V_{sp} = \rho_{sp} \cdot \frac{4}{3} \cdot \pi \cdot \left(\frac{d_{sp}}{2} \cdot 10^{-4}\right)^3$$

The estimated weight was 0.7 pg per spore.

Determination of the Enantiomeric Ratio (*E*-Value) for the product

The Enantiomeric Ratio E was determined according to equation S2, with c: conversion and ee_P: enantiomeric excess of product (P).

$$\text{(S2) } E = \frac{\ln(1-c(1+ee_P))}{\ln(1-c(1-ee_P))}$$

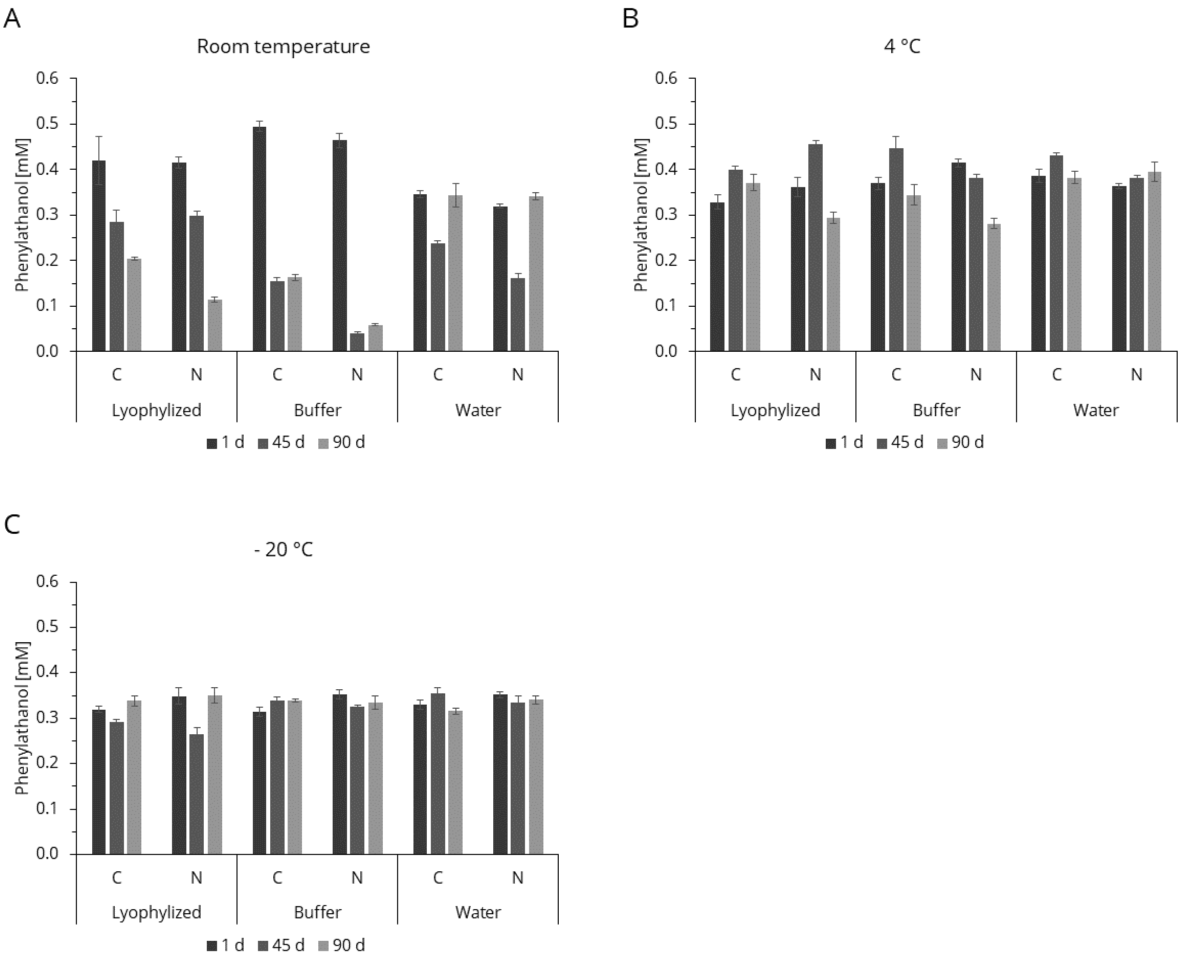

Fig S2 Hydrolytic activity of C- and N-terminally fused SilE-R on Sporobeads with respect to TMS-PhE hydrolysis after storage at different temperatures either dry, in water or in buffer. Spores were stored for 1, 45 and 90 days. Shown are the mean values and standard deviation of the total PhE concentration (A) measured for SilE-R on spores stored at RT in different states. (B) measured for SilE-R on spores stored at 4 °C in various states (C) measured for SilE-R on spores stored at -20 °C in various states. Assay: 4 h duration, 250 µL with 4.6 mmol TMS-PhE L^{-1}, 50 mmol Tris/HCl L^{-1} (pH 8), 0.5 mg spores.
